# Long-Term Follow-Up of a Novel Surgical Option Combining Fibula Free Flap and 3D-Bioprinted, Patient-Specific Polycaprolactone (PCL) Implant for Mandible Reconstruction

**DOI:** 10.3390/bioengineering10060684

**Published:** 2023-06-04

**Authors:** Bo-Yeon Hwang, Kwantae Noh, Jung-Woo Lee

**Affiliations:** 1 Department of Oral and Maxillofacial Surgery, Korea University Anam Hospital, Seoul 02447, Republic of Korea; bo0426@kumc.or.kr; 2 Department of Prosthodontics, School of Dentistry, Kyung Hee University, Seoul 02447, Republic of Korea; nhokt@naver.com; 3 Department of Oral and Maxillofacial Surgery, School of Dentistry, Kyung Hee University, Seoul 02447, Republic of Korea

**Keywords:** mandible reconstruction, PCL, surgical guides, CAD/CAM, bioprinting, patient-specific implant (PSI)

## Abstract

As the fibula free flap became the gold standard in mandibular reconstruction that required both hard tissue and soft tissue, various methods have been sought to solve the height discrepancy between the mandible and fibula. The purpose of this paper was to propose a surgical option that combined the microvascular fibula free flap with a 3D-bioprinted, patient-specific polycaprolactone (PCL) implant as a safe and simple novel method that achieved the best functional and aesthetic results in mandibular reconstruction surgery for young patients with malignant tumors. The patient’s reconstructed mandible maintained volume symmetry without any deformation or complications for over 6 years. Computer-aided design/computer-aided manufacturing (CAD/CAM) and 3D printing technology enabled accurate and safe surgical results.

## 1. Introduction

Extensive mandibular defects resulting from malignant tumor resection require the reconstruction of mandibular continuity, taking into account both function and aesthetics [1,2]. In most cases, the osteocutaneous fibular free flap is the first choice because it offers several advantages such as a reliable vascular pedicle, flexibility, sufficient bone quality and length, and the possibility of a two-team approach [1,3]. However, when the neomandible is created using a single layer of fibula bone, the height is usually insufficient compared to the actual mandible, requiring the surgeon to position the segments in a compromised manner while considering aesthetics and function [4]. To overcome this limitation, the proposed double-barrel fibula flap method involves reconstructing the fibular segments by folding the bone into two layers [5,6]. The upper layer can be used to restore function by installing osseointegrated dental implants at a later stage, whereas the lower layer is positioned in accordance with the mandibular border to achieve optimal aesthetics. However, compared to the single-layer surgery, this double-barreling technique is more challenging and requires greater skill to handle and position the bones and pedicle without the de-vascularization of the flap [7,8]. Therefore, the operation time may be longer, and the risks may be higher [7].

The purpose of this paper is to propose a novel surgical option that combines a microvascular fibula free flap with a patient-specific, 3D-bioprinted PCL implant to achieve a simple two-layer reconstruction while eliminating the technical difficulties in double-barreling. To the best of our knowledge, this is the first long term follow-up case to combine an osteocutaneous fibula free flap and 3D-bioprinted PCL implant in mandible reconstruction.

## 2. Methods

**Figure 1:**
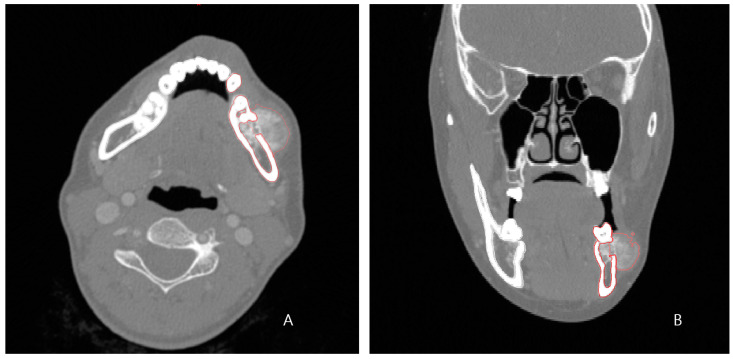
Preoperative enhanced CT (1.0 mm slice) scan showing the malignant tumor eroded the left buccal cortical bone and bulged. (**A**) axial view; (**B**) frontal view (red lines outline the lesion and surrounding tissue).

A 24-year-old female patient was identified with osteosarcoma of the left mandible body and referred to Kyung Hee University Dental Hospital. Preoperative enhanced computed tomography (CT) and enhanced magnetic resonance imaging (MRI) confirmed a tumor of approximately 2.5 cm that had eroded the buccal cortical bone and was bulging out (Figure 1). A wide excision, including segmental mandibulectomy, was planned with a sufficient safety margin. The fibula free flap was considered first since a bony free flap containing soft tissue was required to repair the lining oral mucosa and mandible.

Our patient was a young female, and a more complete reconstruction result was desired without compromising both function and aesthetics, unlike in the case of an elderly patient suffering from a malignant lesion. Additionally, as the tumor was malignant, not benign, a simple and rapid reconstruction operation was necessary to minimize the risk and complications in consideration of the possibility of subsequent treatment. Therefore, considering all of the above, we devised a design that combined the fibular segment and the bio-printed PCL scaffold in two layers instead of the double-barrel method. The fibular portion was placed at the alveolus level for load-bearing and subsequent dental implant insertion, and the PCL scaffold was positioned beneath it to reproduce the patient’s original mandible border contour. This idea was made possible by computer-assisted design/computer-assisted manufacturing (CAD/CAM) and 3D printing technology.

### 2.1. Computer-Assisted Surgical Planning and Design of Patient-Specific Implant

**Figure 2:**
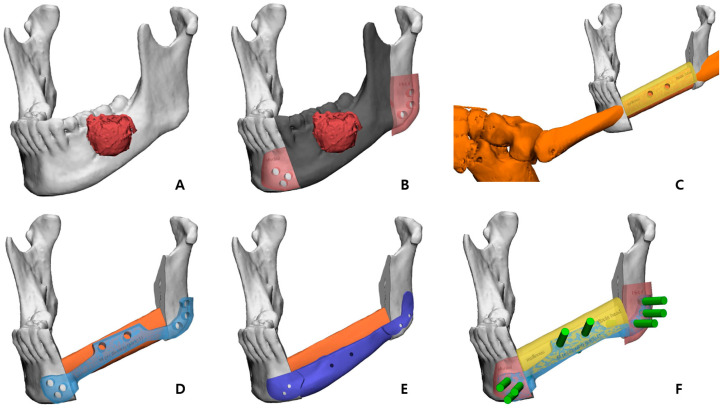
Preoperative 3D simulation for designing surgical guides and patient-specific PCL implant: (**A**) Mandible model generated from the patient’s CT data and tumor (red) isolated from MRI data; (**B**) Resection margin (dark gray) with sufficient safety margin and mandible cutting guides (pink) to reproduce it; (**C**) Left fibula model was moved to the most ideal position at the alveolar level, and the fibula cutting guide (yellow) was designed. (**D**) Fibula positioning guide (sky blue); (**E**) PCL scaffold design (blue) to recreate the mandible border below the fibula segment; (**F**) Fixation holes of all surgical guides are planned to share position vectors (green).

The preoperative patient’s craniomaxillofacial skeleton and lower extremities region-enhanced CT images with slice thicknesses of 1.0 mm were stored in the digital imaging and communication in medical (DICOM) format. These datasets were imported into medical simulation software (Mimics, Materialize NV, Leuven, Belgium) to generate the skull and fibula models through segmentation processing. The standard tessellation language (STL) files of these 3D objects were transferred to medical CAD software (3-matic^®^, Materialize NV, Leuven, Belgium). The mass was extracted by overlapping the enhanced MRI data with the CT data (Figure 2A), and, based on this, the resection part, including the first premolar to the mandibular notch, was determined with a proper safety margin.

**Figure 3:**
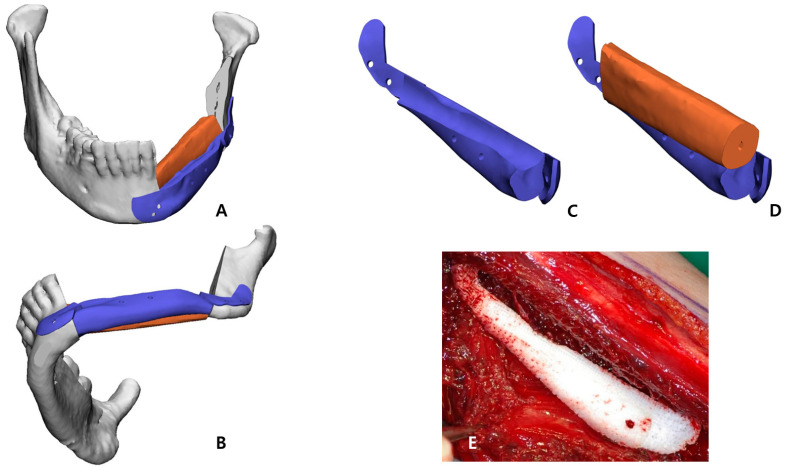
Patient-specific PCL scaffold designed as the lower layer of the fibula segment to ideally regenerate the patient’s mandible border: (**A**) Frontal view; (**B**) Bottom view; (**C**,**D**) Viewed from the inner superior side, it was designed in a concave shape to embrace the upper fibula fragment to increase the accuracy of the position (**E**) Clinical photo of 3D-bioprinted PCL implant with interconnected lattice-type pores.

After performing the planned mandibulectomy on the software, mandible cutting guides were designed to implement it (Figure 2B). The left fibula model was moved to the resection area, and the fibula model was positioned at the alveolar level to facilitate later implant placement, considering the fibula’s shape and pedicle location. To achieve this, the fibula cutting guide and the fibula positioning guide were created (Figure 2C,D). In the inferior part, the scaffold that reproduced the original shape of the patient’s mandibular border was designed to partially cover the outer and lower side of the fibular segment to recreate the volume, and, at the same time, it was intended to support the position of the fibula more accurately (Figure 3). Wing-shaped connection parts with fixation holes that could be fixed to the remaining mandible were added on both sides of the scaffold (Figure 2E). All surgical guides and the scaffold were designed to be fixed with screws through fixation holes, and the locations of these fixation holes were shared to enable reconstruction (Figure 2F).

### 2.2. Fabrication of Surgical Guides and 3D-Bioprinted PCL Implant

The STL file of the PCL implant was sent to a bioprinting company (T&R Biofab Co. Ltd., Seoul, Republic of Korea), which produced it using medical-grade PCL (Evonik Industries, Essen, Germany) in Biofab’s 3D printing system. The PCL raw material was placed within a 10 mL steel syringe for printing. The PCL was heated to 120 °C, which was maintained for the printing duration. The molten PCL was then extruded through 500 µm steel nozzles. The printing code was generated by CAM software. The code was served to slice the customized 3D models and generated a moving path for the 3D printer to operate. The PCL scaffold was then fabricated with interconnected lattice-type pores (Figure 3E). This entire process was performed in accordance with a facility with Good Manufacturing Practice (GMP) certification approved by the MFDS of Korea, and the final fabricated implant was prepared by gamma sterilization.

The surgical guides that we designed were directly printed using our own 3D printer (Form2, Formlabs, Somerville, MA, USA) with clear resin (Formlabs, Somerville, MA, USA), and these were sterilized by the hospital system.

### 2.3. Surgical Technique

**Figure 4:**
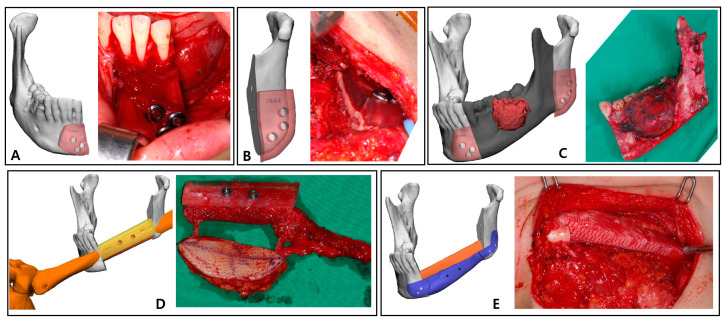
Intraoperative clinical photographs corresponding to 3D simulation: (**A**) Right mandible cutting guide fixed with screws; (**B**) Segmental mandibulectomy performed using the left mandible cutting guide; (**C**) Tumor resected according to the virtual plan using surgical guides; (**D**) Skin paddle, peroneal pedicle, and the fibula segment with fibula cutting guide can be seen in the harvest fibula free flap. (**E**) Three-dimensional-bioprinted PCL implant, with grid pattern recreating mandible border.

The left mandible and tumor were minimally exposed through simultaneous access to the neck and oral cavity without lip splitting. The transparent mandible cutting guides were adapted to the intended location, drilled in accordance with the fixing holes, and secured with screws (Figure 4A,B). The holes generated in the residual mandible were also planned to be used to attach the PCL implant and the fibula positioning guide in the subsequent process. The osteotomy was completed according to the mandible cutting guides, and the mandibular fragment containing the mass was removed in one-bloc (Figure 4C).

The osteofasciocutaneous fibula free flap with a fusiform skin paddle was obtained from the left lower leg by conventional lateral access. Before separating the vascular pedicle, the fibula cutting guide was precisely positioned on the outer surface of the fibula, drilled along the fixation holes, and monocortically fastened with screws. Then, piezoelectric osteotomy was elaborately implemented according to this fibula cutting guide, and the fibula free flap was harvested (Figure 4D). Immediately after this stage, the bio-printed PCL implant was briefly placed in the extracted fibular space to soak in blood containing bone marrow (Figure 3E).

After placing the fibula segment on the defect of the mandible, the fibula positioning guide was accurately fixed with screws using holes already formed in the remaining mandible and fibula segment. In this state, the fibula segment and the remnant mandible were connected with mini-plates and mini-screws. The positioning guide was eliminated, and the patient-specific PCL implant was arranged below the fibula segment (Figure 4E). This scaffold was also fixed with screws using existing holes of the residual mandible. After microvascular anastomosis in the left neck area, the skin paddle regenerated an oral mucosa, and the neck region was closed.

### 2.4. Density and Accuracy Analysis Based on CT Data

**Figure 5:**
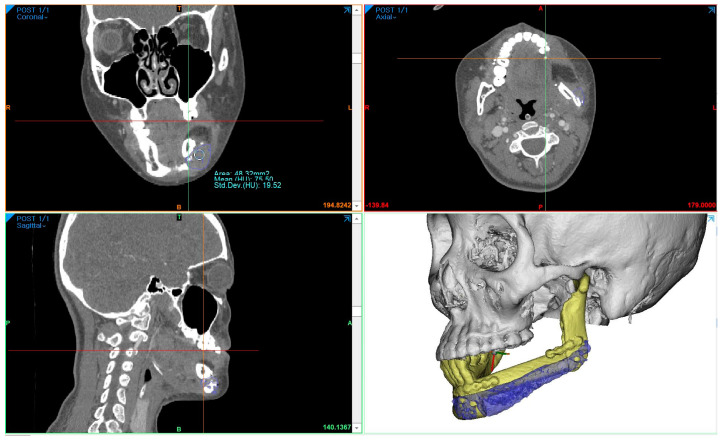
Using the “density” function in Mimics software, the Hounsfield units’ value of the PCL implant area was obtained at the same location. In this case, the measurement of the same region was conducted at the level determined by the location of the hemoclip below the fibula segment and the maxillary first molar in the CT coronal view.

To measure the density of the PCL implant using follow-up-enhanced CT and Mimics software, the same location where the PCL was positioned at the level of the maxillary first molar was identified in the coronal view. At this specific location, the mean value and standard deviation of the Hounsfield units (HUs) were obtained using the “density” function (Figure 5). To compare accuracy on the same software, 3D models of surgical planning and the 3D model generated from the CT data taken two weeks after the operation were superimposed, and the error was calculated through mapping analysis.

## 3. Results

The patient was discharged without any complications after surgery, and the histopathological findings showed chondroblastic osteosarcoma, grade IV, with tumor-free margins. She underwent adjuvant chemotherapy with six cycles of high-dose methotrexate postoperatively and was followed up for over 6 years without metastasis, recurrence, or complications.

**Figure 6:**
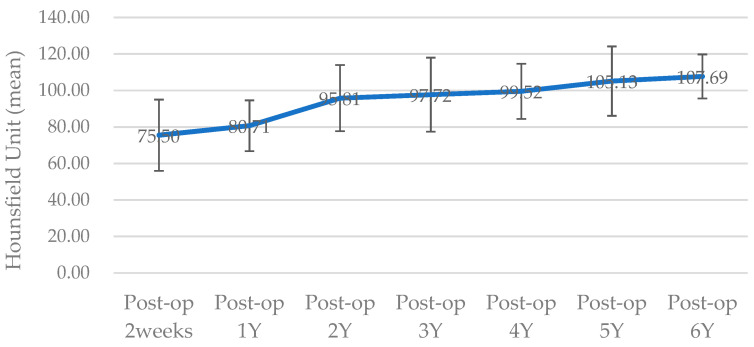
The line chart illustrates the Hounsfield units (HUs) as an indicator of PCL implant density. All HU values were obtained from the same site on CT images collected at follow-up each year (Y) postoperatively (Post-op). The values on the chart represent the mean, whereas the error bars represent the standard deviation (SD).

**Figure 7:**
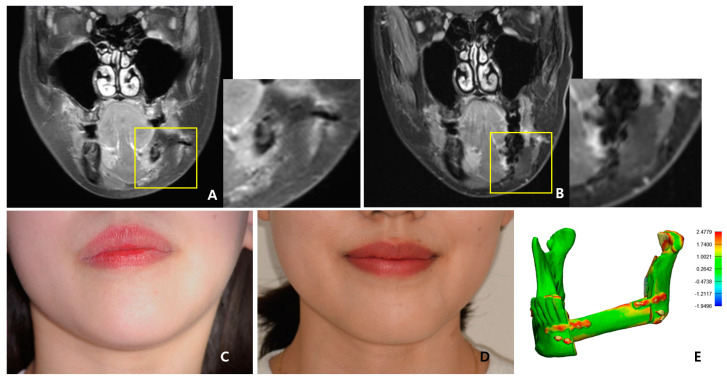

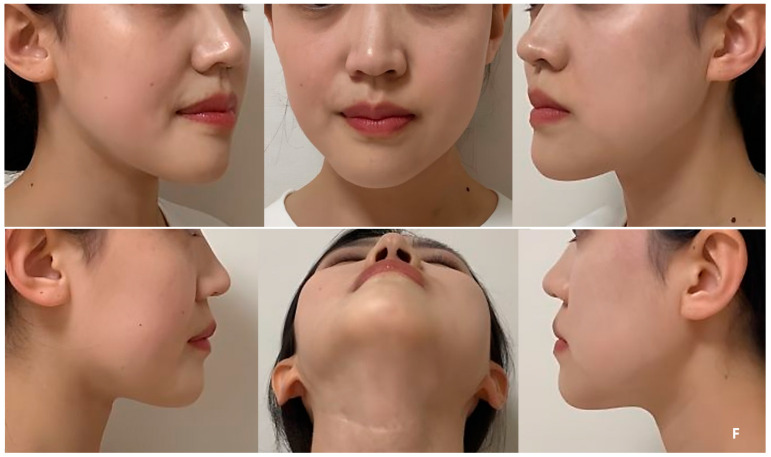
Postoperative findings: (**A**) The lattice pattern of the PCL was clearly visible on the MRI coronal at 11 months postoperative, and (**B**) the pattern was not seen on MRI coronal at the 40 months postoperative. The yellow box here presents the area containing the PCL implant, which has been magnified and displayer in the adjacent image. As shown in (**C**) the preoperative photograph and (**D**) the 6−year postoperative photograph, the volume of the patient’s mandible has been maintained symmetrically. (**E**) As a result of comparative mapping by superimposition of the virtual plan and postoperative CT data, an error within 1 mm was observed. (**F**) Clinical photographs from multiple angles at 6 years and 4 months postoperative; the symmetrical contour of the mandible was confirmed.

The surgical results were analyzed using enhanced CT and MRI, which were radiographic images taken for the purpose of tracking malignant tumors. Follow-up CT scans were conducted at 2 weeks postoperatively and every year for the next 6 years, whereas MRI scans were performed only at 5 months, 11 months, 40 months, and 4-and-a-half years after surgery. The PCL implant exhibited similar attenuation to the surrounding soft tissue on the enhanced CT images, making it challenging to accurately generate a 3D model rendering in the Mimics software. The Hounsfield unit value of the PCL implant was measured using the “density” function in this software. It was observed that the density progressively increased over the years, from 75.50 at 2 weeks after surgery to 107.69 at 6 years after surgery (Figure 6). All average values were within the range of muscle thresholds. On the follow-up MRI, the PCL implant exhibited signals between the surrounding soft tissue and hard tissue, allowing for clear margin distinction. Consequently, changes in volume could be observed on the MRI, and there were no significant changes until 4-and-a-half years after surgery. The lattice pattern of the PCL, which was clearly observed until the 11-month postoperative MRI image, was absent in the 40-month postoperative MRI image, and it was analyzed that only the surface of the PCL had been replaced with soft tissue (Figure 7A,B). By superimposing the model immediately after surgery with the virtual plan, it was determined that the error was within 1 mm, except for the condylar head (Figure 7E).

As shown in the series of facial clinical photos, the patient’s surgical site has been maintaining volume symmetry without any deformation for over 6 years, and the patient has been satisfied with her appearance (Figure 7C,D,F). During each follow-up, upon palpation of the reconstructed region, it was confirmed that the PCL implant area was firmly maintained and smoothly connected to the mandible without any irregular edges.

**Figure 8:**
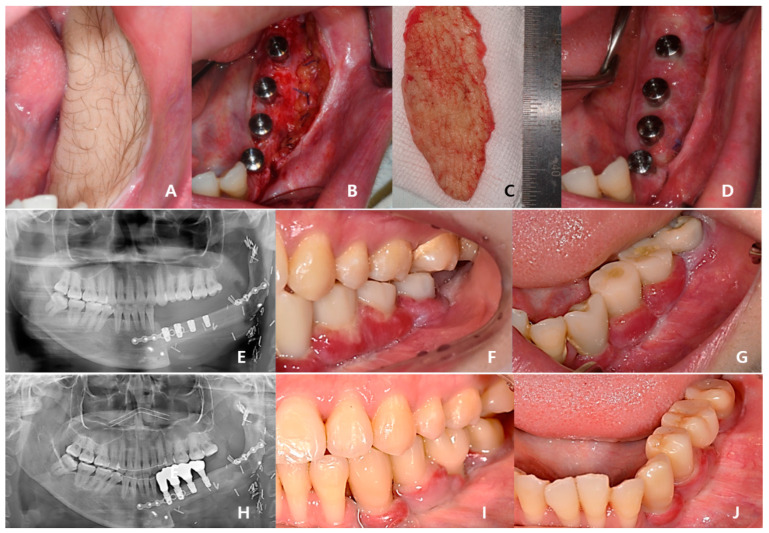
In total, 40 months after cancer surgery, the secondary implant surgery was performed, and the surrounding mucosa was sutured to the periosteum of the fibula to form an attached gingiva: (**A**) preoperative intraoral photo with hairy fibular skin; (**B**) postoperative intraoral photo; (**C**) removed fibular skin; (**D**) stich-out after 2 weeks; (**E**) panoramic view immediately after dental implant 1st surgery; (**F**,**G**) temporary prosthesis after 4 months; (**H**–**J**) Recovery of masticatory function was completed without inflammation after delivery of the final prosthesis 4 years after cancer surgery.

Three years after cancer surgery, 2-stage dental implant (TS-III CA; Osstem Implant Co., Busan, Republic of Korea) installation was performed outpatiently under local anesthesia, the final implant prosthesis was completed 4 years after surgery, and the patient recovered functionally and aesthetically (Figure 8).

## 4. Discussion

The purpose of this paper was to propose a surgical option that combined a microvascular fibula free flap with a patient-specific PCL implant as a safe and simple novel method that achieved the best results both functionally and aesthetically in mandible reconstruction surgery for young patients with malignant tumors.

As the fibula free flap became the gold standard in mandibular reconstruction that required soft tissue and hard tissue simultaneously, various methods have been sought to solve the height discrepancy between the mandible and fibula, which was almost the only limitation [4,9]. These included the double-barrel fibula, the use of an additional inferior reconstruction plate, and vertical distraction osteogenesis [4,5,10,11]. After Horiuchi et al. proposed the double-barrel fibula graft for mandibular reconstruction in 1995, several successful results of the method have been published [6]. In particular, with the recent development of 3D simulation CAD/CAM and 3D printing technology, more diverse attempts have become possible [3,12,13]. Yang et al. produced a design that increased the accuracy in the positioning of two-strut fibula segments by fabricating a “one-piece” patient-specific plate [14]. Berrone et al. used long dental implants to secure the double-barrel segments and fixed only the lower layer to the mandible using a patient-specific plate [15]. Lee et al. reproduced the planned position through virtual surgery by fixing the double-barrel fibula with the positioning guide that shared screw holes and then performed fixation using a mini-plate in that state to ensure accuracy [16]. Although the problem of difficulty in precisely locating multiple bone segments as mentioned above could be overcome with technology, the double-barrel fibula graft was still complicated and risky due to the fundamental limitation of folding the pedicle [7].

In order to overcome the restriction of the double-barrel fibula graft and enable two-strut reconstruction [17], Lee et al. proposed a hybrid graft method with a non-vascularized residual fibula bone [7]. Since then, various designs have been tried that placed non-vascularized fibula segments only in areas necessary for dental implant placement. Although these options simplified the operation, there were cases where the non-vascular part was resorbed, and bone formation was not possible [18]. Therefore, to prevent the possibility of resorption of the site where dental implants were to be inserted, we placed the vascular fibula segment at the alveolar level so that the autogenous bone continued and located the patient-specific PCL implant below that reproduced the original shape of the mandible. This surgical option for two-layer reconstruction was simple, safe, and provided an ideal outcome.

Polycaprolactone (PCL) is an FDA-approved, biocompatible, and biodegradable safety biomaterial that has been studied as a drug delivery device since the 1970s [19,20]. Recently, it has been used not only to prevent airway stenosis in infants with tracheobronchomalacia but also to be applied for the reconstruction of maxillary, infraorbital, and cranial defects as a patient-specific, 3D-printed scaffold [21,22,23]. According to the finite element analysis of mandible reconstruction, PCL alone cannot withstand the masticatory force, and the wing design helps to distribute the stress [24,25]. Our design also did not use PCL alone; we placed it in the lower part, unrelated to the bearing of the masticatory force, and added the wing design to disperse stress. This wing part shared the holes with the drilling holes of surgical guides so that the PCL implant could be secured in the planned position. Additionally, the PCL implant was designed to cover the inferior surface of the fibula bone, which had the benefit of guiding the location of the fibula part.

Han et al. applied a patient-specific PCL scaffold to reconstruct a complex maxillary defect and reported stable and aesthetic surgical results [21]. The Hounsfield unit values of CT images at 6 months and 16 months after surgery were compared and analyzed as an increase in scaffold density. However, even on enhanced CT, the PCL part was distinguishable from the hard tissue but had a similar attenuation to the surrounding soft tissues. As a result, it was impossible to clearly demarcate the margin of the PCL, and only the extent of its position could be grasped. Therefore, in order to objectively compare the density of the PCL, HUs measured at the same location were required. In our case, the maxillary tooth and surrounding hemoclip in a fixed position were used as references. The HU values were acquired using the “density” function of the Mimics software. These values increased gradually each year, with all falling within the range of HU values for muscle. This observation suggested that the biodegradable PCL was replaced by soft tissues with a density similar to that of muscle, leading to an overall increase in density. Jeong et al. reported accurate and esthetic surgical outcomes of eight patients in zygomatico-maxillary reconstruction using a PCL/beta tricalcium phosphate (β-TCP) scaffold [22]. In their analysis of the results based on CT, a volume-rendering 3D model was created for the postsurgical implant to calculate the volume increase, and the HU values were measured to report the rate of new bone formation. However, due to the similar attenuation of the PCL implant to the nearby soft tissue, it was not possible to generate a volume-rendering model of the PCL implant alone in CT through segmentation. In contrast, on the MRI, the PCL implant showed intermediated signals between the hard tissue and the soft tissue; thus, it was possible to establish the boundary between the surrounding soft tissues. Even the PCL implant with interconnected lattice-type pores was observed as a lattice pattern on the MRI image. This grid pattern, which was clearly seen on MRI at 5 and 11 months after surgery, was not observed from MRI at 40 months postoperatively. Therefore, volume comparison of the PCL was possible with MRI, not CT. When comparing the latest follow-up MRI images taken 4-and-a-half years postoperatively, with previous MRI images, it was observed that the volume of the patient’s PCL implant itself did not change significantly. This finding was in contrast to reports of active disassembly occurring at 2 to 3 years in general conditions [19,20,26]. Additionally, it was seen that the surface of the PCL implant was replaced with soft tissue, having a signal similar to that of the muscle. Based on the fact that PCL is a biodegradable material, the above results could be interpreted as not regenerating into bone tissue at the resolved site but being replaced by the rigid soft tissue homogeneous to the muscle, so that the patient’s facial deformation may not occur.

Three-dimensional, metal-printed, patient-specific implants (PSIs) have been attempted as replacements for bone flaps in mandible reconstruction, and titanium (Ti) is preferred mainly due to its advantages such as its high tensile strength and osseointegration properties [27,28,29]. Oassemar et al. reported that the application of titanium PSIs in mandible reconstruction is an alternative method, especially when the patient has poor general condition, previous surgery at the same site, radiation treatment, or a donor site vascular defect; thus, microsurgery is not possible [30]. In addition, several cases of applying Ti PSIs to correct the asymmetry of the mandible have been reported [29,31,32]. However, titanium facial implants continue to have problems such as extrusion, migration, foreign body reaction, and infection that alloplastic implants can have [32,33]. In particular, if dental implants are connected for masticatory function, they become more vulnerable to infection [33]. Due to the enormous production cost and long production period, the patient’s economic burden is high, and there is a time limit in applying it to malignant patients; thus, there is still a limit to general use. In contrast, biodegradable PCL can overcome the restrictions of exposure, inflammation, and infection that metal implants have, and is less burdensome with a relatively shorter manufacturing period and lower cost than them [19]. In order for PCL, which has these advantages, to be applied as a substitute for bone flaps in mandible reconstruction, bone generation must be possible, and additional research on this is needed [21]. Additionally, for a clinical analysis, it seems necessary to have an attenuation that distinguishes PCL on CT images.

CAD/CAM and 3D printing technology ensure the accuracy of reconstruction, reduce operation time and ischemia time, and, eventually, improve surgical results [3,13,15,34]. Tack et al. reported in a literature review of 3D printing that it is applied in multiple surgical domains because of the above benefits, and there are many related papers in the order of surgical guides, surgical planning, and custom implants [12]. Our surgical design utilizes all of the above techniques, and, as a result, accurate and safe surgery can be performed. In addition, the long-term stable clinical results of this case for over 6 years lead to considering the primary application of the surgical option in mandibular reconstruction for young patients. These patients are a target group that requires satisfying aesthetics and functions to a relatively higher degree. The biocompatible PCL implant has no absolute contraindications, although it may not be feasible for patients who cannot undergo the fibula free flap procedure mentioned earlier [30], which is rare among young patients. To expand its applicability beyond young patients, it is necessary to reduce production time and cost. As the patient is tumor-free and has completed the 5-year follow-up period for malignant tumors, MRI and CT scans are not required. However, follow-ups of the reconstructed sites and dental implants will continue, allowing for data reporting over a longer period of time.

## 5. Conclusions

This new surgical option, which combines the free fibula flap with a 3D-bio-printed PCL, ensures optimal aesthetic results while guaranteeing function in mandibular reconstruction. A patient-specific PCL implant that shares the screw holes of surgical guides improves the accuracy, simplifies the operation, reduces the operation time, and, consequently, enables safe surgical outcomes. Through long-term follow-up, we have shown that this method produces ideal surgical results and remains stable. It is expected that these positive outcomes will lead to considering the primary application of the combined design in the mandibular reconstruction of young patients.

## Data Availability

Not applicable.

## References

[1] Cordeiro P.G., Disa J.J., Hidalgo D.A., Hu Q.Y. (1999). Reconstruction of the mandible with osseous free flaps: A 10-year experience with 150 consecutive patients. Plast. Reconstr. Surg..

[2] Hidalgo D.A. (1989). Fibula free flap: A new method of mandible reconstruction. Plast. Reconstr. Surg..

[3] Hirsch D.L., Garfein E.S., Christensen A.M., Weimer K.A., Saddeh P.B., Levine J.P. (2009). Use of computer-aided design and computer-aided manufacturing to produce orthognathically ideal surgical outcomes: A paradigm shift in head and neck reconstruction. J. Oral. Maxillofac. Surg..

[4] Su T., Fernandes R. (2014). Microvascular reconstruction of the mandible: An argument for the fibula osteocutaneous free flap. Rev. Española De Cirugía Oral Y Maxilofac..

[5] Bähr W., Stoll P., Wächter R. (1998). Use of the “double barrel” free vascularized fibula in mandibular reconstruction. J. Oral Maxillofac. Surg..

[6] Horiuchi K., Hattori A., Inada I., Kamibayashi T., Sugimura M., Yajima H., Tamai S. (1995). Mandibular reconstruction using the double barrel fibular graft. Microsurgery.

[7] Lee J., Kim M., Choi W., Yoon P., Ahn K., Myung H., Hwang S., Seo B., Choi J., Choung P. (2004). Concomitant reconstruction of mandibular basal and alveolar bone with a free fibular flap. Int. J. Oral Maxillofac. Surg..

[8] Yang W.F., Zhang C.Y., Choi W.S., Zhu W.Y., Li D.T.S., Chen X.S., Du R., Su Y.X. (2020). A novel ‘surgeon-dominated’ approach to the design of 3D-printed patient-specific surgical plates in mandibular reconstruction: A proof-of-concept study. Int. J. Oral. Maxillofac. Surg..

[9] Wallace C.G., Chang Y.-M., Tsai C.-Y., Wei F.-C. (2010). Harnessing the potential of the free fibula osteoseptocutaneous flap in mandible reconstruction. Plast. Reconstr. Surg..

[10] Nocini P.F., Wangerin K., Albanese M., Kretschmer W., Cortelazzi R. (2000). Vertical distraction of a free vascularized fibula flap in a reconstructed hemimandible: Case report. J. Cranio-Maxillofac. Surg..

[11] Marchetti C., Bianchi A., Mazzoni S., Cipriani R., Campobassi A. (2006). Oromandibular reconstruction using a fibula osteocutaneous free flap: Four different “preplating” techniques. Plast. Reconstr. Surg..

[12] Tack P., Victor J., Gemmel P., Annemans L. (2016). 3D-printing techniques in a medical setting: A systematic literature review. Biomed. Eng. Online.

[13] Gil R.S., Roig A.M., Obispo C.A., Morla A., Pagès C.M., Perez J.L. (2015). Surgical planning and microvascular reconstruction of the mandible with a fibular flap using computer-aided design, rapid prototype modelling, and precontoured titanium reconstruction plates: A prospective study. Br. J. Oral Maxillofac. Surg..

[14] Yang W.F., Choi W.S., Zhu W.Y., Su Y.X. (2020). “One-piece” patient-specific reconstruction plate for double-barrel fibula-based mandibular reconstruction. Int. J. Oral. Maxillofac. Surg..

[15] Berrone M., Crosetti E., Battiston B., Succo G. (2020). Virtual surgical planning for mandible reconstruction with a double barrel fibula flap and immediate implant placement. J. Craniofacial Surg..

[16] Lee J.W., Choi B.J., Lee D.W., Kwon Y.D. (2016). Double-barrelled vascularised fibular free flap using computer-assisted preoperative planning and a surgical template for accurate reconstruction of a segmental mandibular defect. Br. J. Oral. Maxillofac. Surg..

[17] Lin B., Yang H., Yang H., Shen S. (2019). Vascularized combined with nonvascularized fibula flap for mandibular reconstruction: Preliminary results of a novel technique. J. Craniofacial Surg..

[18] Pogrel M., Podlesh S., Anthony J.P., Alexander J. (1997). A comparison of vascularized and nonvascularized bone grafts for reconstruction of mandibular continuity defects. J. Oral. Maxillofac. Surg..

[19] Woodruff M.A., Hutmacher D.W. (2010). The return of a forgotten polymer—Polycaprolactone in the 21st century. Prog. Polym. Sci..

[20] Yeo A., Rai B., Sju E., Cheong J., Teoh S. (2008). The degradation profile of novel, bioresorbable PCL–TCP scaffolds: An in vitro and in vivo study. J. Biomed. Mater. Res. Part A Off. J. Soc. Biomater. Jpn. Soc. Biomater. Aust. Soc. Biomater. Korean Soc. Biomater..

[21] Han H.H., Shim J.-H., Lee H., Kim B.Y., Lee J.-S., Jung J.W., Yun W.-S., Baek C.H., Rhie J.-W., Cho D.-W. (2018). Reconstruction of complex maxillary defects using patient-specific 3D-printed biodegradable scaffolds. Plast. Reconstr. Surg. Glob. Open.

[22] Jeong W.-S., Kim Y.-C., Min J.-C., Park H.-J., Lee E.-J., Shim J.-H., Choi J.-W. (2022). Clinical application of 3D-printed patient-specific polycaprolactone/beta tricalcium phosphate scaffold for complex zygomatico-maxillary defects. Polymers.

[23] Morrison R.J., Hollister S.J., Niedner M.F., Mahani M.G., Park A.H., Mehta D.K., Ohye R.G., Green G.E. (2015). Mitigation of tracheobronchomalacia with 3D-printed personalized medical devices in pediatric patients. Sci. Transl. Med..

[24] Prasadh S., Suresh S., Hong K.L., Bhargav A., Rosa V., Wong R.C.W. (2020). Biomechanics of alloplastic mandible reconstruction using biomaterials: The effect of implant design on stress concentration influences choice of material. J. Mech. Behav. Biomed. Mater..

[25] Lee S., Choi D., Shim J.-H., Nam W. (2020). Efficacy of three-dimensionally printed polycaprolactone/beta tricalcium phosphate scaffold on mandibular reconstruction. Sci. Rep..

[26] Sun H., Mei L., Song C., Cui X., Wang P. (2006). The in vivo degradation, absorption and excretion of PCL-based implant. Biomaterials.

[27] Owusu J.A., Boahene K. (2015). Update of patient-specific maxillofacial implant. Curr. Opin. Otolaryngol. Head Neck Surg..

[28] Horta R., Frias F., Jarnalo M., Teixeira S., Silva P., Oliveira I., Silva A. (2020). Facial reconstruction based on combined three-dimensional printing and microsurgical free transfer. J. Craniofacial Surg..

[29] Yates J.M., Wildgoose D.G., van Noort R. (2009). Correction of a mandibular asymmetry using a custom-made titanium onlay. J. Plast. Reconstr. Aesthetic Surg..

[30] Qassemyar Q., Assouly N., Temam S., Kolb F. (2017). Use of a three-dimensional custom-made porous titanium prosthesis for mandibular body reconstruction. Int. J. Oral Maxillofac. Surg..

[31] Lee U.-L., Kwon J.-S., Woo S.-H., Choi Y.-J. (2016). Simultaneous bimaxillary surgery and mandibular reconstruction with a 3-dimensional printed titanium implant fabricated by electron beam melting: A preliminary mechanical testing of the printed mandible. J. Oral Maxillofac. Surg..

[32] Watson J., Hatamleh M., Alwahadni A., Srinivasan D. (2014). Correction of facial and mandibular asymmetry using a computer aided design/computer aided manufacturing prefabricated titanium implant. J. Craniofacial Surg..

[33] Kim H.J., Moon S.Y. (2020). The deep circumflex iliac artery flap for mandibular reconstruction and donor site reconstruction with a patient-specific implant: A case report. Appl. Sci..

[34] AlAli A.B., Griffin M.F., Butler P.E. (2015). Three-dimensional printing surgical applications. Eplasty.

